# Alignment-free sequence comparison: benefits, applications, and tools

**DOI:** 10.1186/s13059-017-1319-7

**Published:** 2017-10-03

**Authors:** Andrzej Zielezinski, Susana Vinga, Jonas Almeida, Wojciech M. Karlowski

**Affiliations:** 1 Department of Computational Biology, Faculty of Biology, Adam Mickiewicz University in Poznan, Umultowska 89, 61-614 Poznan, Poland; 2 IDMEC, Instituto Superior Técnico, Universidade de Lisboa, Av. Rovisco Pais 1, 1049-001 Lisbon, Portugal; 3 Stony Brook University (SUNY), 101 Nicolls Road, Stony Brook, NY 11794 USA

## Abstract

Alignment-free sequence analyses have been applied to problems ranging from whole-genome phylogeny to the classification of protein families, identification of horizontally transferred genes, and detection of recombined sequences. The strength of these methods makes them particularly useful for next-generation sequencing data processing and analysis. However, many researchers are unclear about how these methods work, how they compare to alignment-based methods, and what their potential is for use for their research. We address these questions and provide a guide to the currently available alignment-free sequence analysis tools.

**Electronic supplementary material:**

The online version of this article (doi:10.1186/s13059-017-1319-7) contains supplementary material, which is available to authorized users.

## Introduction

The 1980s and 1990s were a flourishing time not only for pop music but also for bioinformatics, where the emergence of sequence comparison algorithms revolutionized the computational and molecular biology fields. At that time, many computational biologists quickly became stars in the field by developing programs for sequence alignment, which is a method that positions the biological sequences’ building blocks to identify regions of similarity that may have consequences for functional, structural, or evolutionary relationships. Many successful alignment-based tools were created including sequence similarity search tools (e.g., BLAST [1], FASTA [2]), multiple sequence aligners (e.g., ClustalW [3], Muscle [4], MAFFT [5]), sequences’ profile search programs (e.g., PSI-BLAST [1], HMMER/Pfam [6]), and whole-genome aligners (e.g., progressive Mauve [7], BLASTZ [8], TBA [9]); these tools became game-changers for anyone who wanted to assess the functions of genes and proteins.

All alignment-based programs, regardless of the underlying algorithm, look for correspondence of individual bases or amino acids (or groups thereof) that are in the same order in two or more sequences. The procedure assumes that every sequence symbol can be categorized into at least one of two states—conserved/similar (match) or non-conserved (mismatch)—although most alignment programs also model inserted/deleted states (gaps). However, as our understanding of complex evolutionary scenarios and our knowledge about the patterns and properties of biological sequences advanced, we gradually uncovered some downsides of sequence comparisons based solely on alignments.

## Five cases where alignment-based sequence analysis might be troublesome

First, alignment-producing programs assume that homologous sequences comprise a series of linearly arranged and more or less conserved sequence stretches. However, this assumption, which is termed collinearity, is very often violated in the real world. A good example is viral genomes, which exhibit great variation in the number and order of genetic elements due to their high mutation rates, frequent genetic recombination events, horizontal gene transfers, gene duplications, and gene gains/losses [10]. These large-scale evolutionary processes essentially occur all the time in the genomes of other organisms. As a result, each genome becomes a mosaic of unique lineage-specific segments (i.e., regions shared with a subset of other genomes). Furthermore, the alignment approach may often overlook rearrangements on an even smaller scale; for instance, the linear and modular organization of proteins is not always preserved due to frequent domain swapping, or duplication or deletion of long peptide motifs [11, 12].

Second, the accuracy of sequence alignments drops off rapidly in cases where the sequence identity falls below a certain critical point. For protein sequences, there are 20 possible amino acid residues, and any two unrelated sequences can match at up to 5% of the residues. If gaps are allowed, then the percentage can increase to 25% [13]. Thus, in practical applications, the area of 20–35% identity is commonly regarded as the “twilight zone” [14], where remote homologs mix with random sequences. Below 20% identity, in the realm of the “midnight zone”, homologous relationships cannot be reliably determined with plain pairwise alignments, often requiring more sophisticated alignment-based solutions, like profiles (e.g., PSI-BLAST) and hidden Markov models (e.g., HMMER). This failure is especially problematic in the annotation of protein superfamilies where the members retain structural kinship even when the average intersequence identity is 8–10% [15]. For nucleotide sequences, the accuracy of the alignments is even more disappointing. For instance, two randomly related DNA/RNA sequences can show up to 50% sequence identity when gaps are allowed, and the edge of the twilight zone can encompass nucleotide matches of up to 60–65% [16–18].

Third, alignment-based approaches are generally memory consuming and time consuming and thus are of limited use with multigenome-scale sequence data. The number of possible alignments of two sequences grows rapidly with the length of the sequences (for two sequences of length *N* there are (2 *N)*!*/(N*!*)* ^2^ different gapped alignments [19], which results in about 10^60^ alignments for two sequences of length 100). Although there is a method, called dynamic programming, that guarantees obtaining a mathematically optimal (highest scoring) alignment without listing all possible solutions, it is also computationally demanding (time complexity is in the order of the product of the lengths of the input sequences) [20]. Therefore, despite the wealth of tools and more than 15 years of research [7, 21–25], the problem of long sequence alignment is not fully resolved [26]. In addition, available sequence evolutionary models may not directly apply to complete genomes, as recently implicated by the Alignathon project, where over 50% of the aligned positions—at the nucleotide level—were inconsistent between pairs of 13 tested methods [26]. Therefore, even the designers of the alignment algorithms and browsers do not claim that their results are correct at all sites across entire genomes [27].

Fourth, the computation of an accurate multiple-sequence alignment is an NP-hard problem, which means that the alignment cannot be solved in a realistic time frame. This situation explains why more than 100 alternative faster methods have been developed over the past three decades [28]. However, the speed optimization does not come without “cost”. These techniques rely on various shortcuts (heuristics) that do not guarantee the identification of the optimal and highest scoring alignment and often result in inaccuracies that limit the quality of many downstream analyses (e.g., phylogenetic). The complexity of the sequence alignment problem even calls for crowdsourcing solutions (e.g., creating the online game Phylo to improve computer-created multiple sequence alignments) [29].

Finally, a sequence alignment depends on multiple a priori assumptions about the evolution of the sequences that are being compared. These various parameters (e.g., substitution matrices, gap penalties, and threshold values for statistical parameters) are somewhat arbitrary, which additionally strains Occam’s razor to breaking point. Moreover, the scoring system is not consensual between applications, and many reports have shown that small changes in the input parameters can greatly affect the alignment [30]. Despite the awareness of the problem, how to choose alignment parameters may often cause problems and usually requires a trial and error approach. (i.e., if an alignment is not good enough, then one can tweak input parameters to get “better-looking” results). Furthermore, reference substitution matrices required for protein alignments (e.g., different series of BLOSUM and PAM) are often used without verifying whether they are representative of the sequences being aligned. Intriguingly, BLOSUM matrices, which are the most commonly used substitution matrix series for protein sequence alignments, were found to have been miscalculated years ago and yet produced significantly better alignments than their corrected modern version (RBLOSUM) [31]; this paradox remains a mystery.

## What is alignment-free sequence comparison?

Alignment-free approaches to sequence comparison can be defined as any method of quantifying sequence similarity/dissimilarity that does not use or produce alignment (assignment of residue–residue correspondence) at any step of algorithm application. From the start, such restriction places the alignment-free approaches in a favorable position—as alignment-free methods do not rely on dynamic programming, they are computationally less expensive (as they are generally of a linear complexity depending only on the length of the query sequence [32]) and therefore suitable for whole genome comparisons [33–36]. Alignment-free methods are also resistant to shuffling and recombination events and are applicable when low sequence conservation cannot be handled reliably by alignment [37]. Finally, in contrast to alignment-based methods, they do not depend on assumptions regarding the evolutionary trajectories of sequence changes. Although these characteristics apply to all alignment-free methods, there are more than 100 techniques to consider [37].

Alignment-free approaches can be broadly divided into two groups [38, 39]: methods based on the frequencies of subsequences of a defined length (word-based methods) and methods that evaluate the informational content between full-length sequences (information-theory based methods). There are also methods that cannot be classified in either of the groups, including those based on the length of matching words (common [40], longest common [41], or the minimal absent [42, 43] words between sequences), chaos game representation [44], iterated maps [45], as well as graphical representation of DNA sequences, which capture the essence of the base composition and distribution of the sequences in a quantitative manner [46, 47].

All of the alignment-free approaches are mathematically well founded in the fields of linear algebra, information theory, and statistical mechanics, and calculate pairwise measures of dissimilarity or distance between sequences. Conveniently, most of these measures can be directly used as an input into standard tree-building software, such as Phylip [48] or MEGA [49].

## How do word frequency-based methods work?

**Fig. 1:**
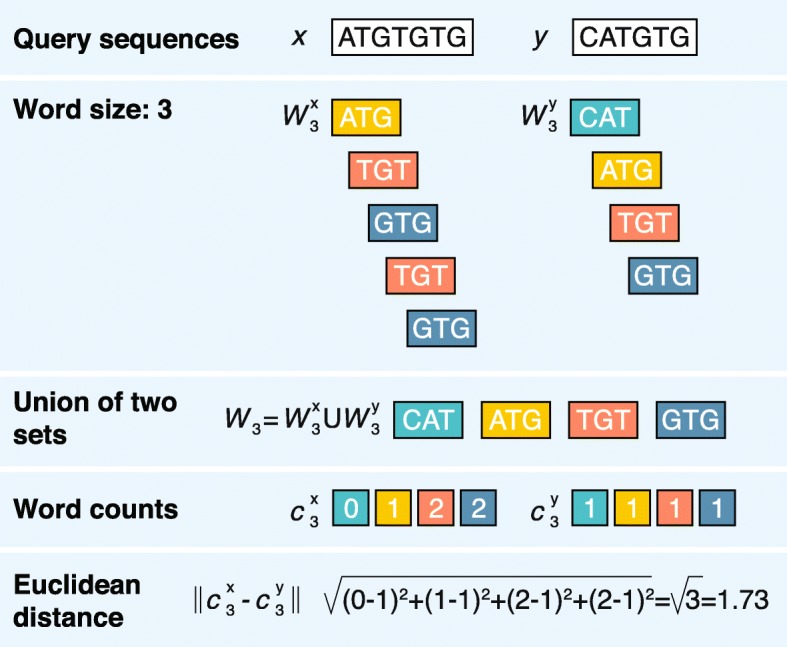
Alignment-free calculation of the word-based distance between two sample DNA sequences ATGTGTG and CATGTG using the Euclidean distance

The rationale behind these methods is simple: similar sequences share similar words/*k*-mers (subsequences of length *k*), and mathematical operations with the words’ occurrences give a good relative measure of sequence dissimilarity. The method is also tightly coupled with the idea of genomic signatures, which were first introduced for dinucleotide composition (e.g., GC content) [50] and further extended to longer words. This process can be broken into three key steps (Fig. 1).

First, the sequences being compared must be sliced up into collections of unique words of a given length. For example, two DNA sequences *x =* ATGTGTG and *y =* CATGTG and a word size of three nucleotides (3-mers) produces two collections of unique words: *W* ^*X*^_*3*_ *=* {ATG, TGT, GTG} and *W* ^*Y*^_*3*_ *=* {CAT, ATG, TGT, GTG}. Because some words are often present in one sequence but not in the other sequence (i.e., CAT in *y* but not in *x*), we create a full set of words that belong to at least *W* ^*X*^_*3*_ or *W* ^*Y*^_*3*_ to further simplify the calculations, resulting in the union set *W*_3_ *=* {ATG, CAT, GTG, TGT}.

The second step is to transform each sequence into an array of numbers (vector) (e.g., by counting the number of times each particular word (from *W*_*3*_) appears within the sequences). For sequences *x* and *y*, we identify two real-valued vectors: *c* ^*X*^_*3*_ *=* (1, 0, 2, 2) and *c* ^*Y*^_*3*_ *=* (1, 1, 1, 1).

The last step includes quantification of the dissimilarity between sequences through the application of a distance function to the sequence-representing vectors *c* ^*X*^_*3*_ and *c* ^*Y*^_*3*_. This difference is very commonly computed by the Euclidean distance, although any metric can be applied [51]. The higher the dissimilarity value, the more distant the sequences; thus, two identical sequences will result in a distance of 0.

Word-based alignment-free algorithms come in different colors and flavors, with methodological variations at each of the three basic steps. In the first step, one can try any resolutions of word lengths—it is important to choose words that are not likely to commonly appear in a sequence (the shorter the word, then the more likely it will appear randomly in a sequence). In practice, the word size (*k*) of 2–6 residues produces stable and optimal protein sequence comparisons across a wide range of different phylogenetic distances [52, 53]; in nucleotide sequence analyses, *k* can safely be set to 8–10 for genes or RNA [54], 9–14 bases for general phylogenetic analyses [34, 55, 56], and up to 25 bases in case of comparison of isolates of the same bacterial species [33, 57]. As a rule of thumb, smaller *k*-mers should be used when sequences are obviously different (e.g., they are not related) whereas longer *k*-mers can be used for very similar sequences [55, 58]. Alternatively, DNA/RNA or protein alphabet can be reduced to a smaller number of symbols based on chemical equivalences. This procedure may increase the detection of homologous sequences that display very low identity [53]. For example, the four-letter DNA alphabet can be distilled to two-letter purine–pyrimidine encoding [55], and proteins can be represented by 5, 4, 3, or even 2 letters according to their different physical–chemical properties [52]. The second step (mapping sequences onto vectors) is by far the most customizable; instead of using vectors of word counts or word frequencies, there are many other ways to create vectors, which range from weighting techniques to normalization and clustering [32]. Additionally, because word-based methods operate on vectors, their mathematical elegance allows the employment of more than 40 functions other than the Euclidean distance, such as the Pearson correlation coefficient [38], Manhattan distance, and Google distance [59].

## How do information theory-based methods work?

Information theory-based methods recognize and compute the amount of information shared between two analyzed biological sequences. Nucleotide and amino acid sequences are ultimately strings of symbols, and their digital organization is naturally interpretable with information theory tools, such as complexity and entropy.

**Fig. 2:**
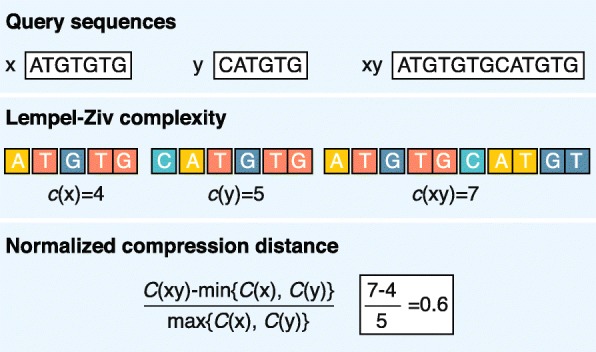
Alignment-free calculation of the normalized compression distance using the Lempel–Ziv complexity estimation algorithm. Lempel–Ziv complexity counts the number of different words in sequence when scanned from left to right (e.g., for *s* = ATGTGTG, Lempel–Ziv complexity is 4: A|T|G|TG). Description of compression algorithms in alignment-free analysis has been reviewed extensively [63]

For example, the Kolmogorov complexity of a sequence can be measured by the length of its shortest description. Accordingly, the sequence AAAAAAAAAA can be described in a few words (10 repetitions of A), whereas CGTGATGT presumably has no simpler description than specification nucleotide by nucleotide (1 C, then 1 G and so on). Intuitively, longer sequence descriptions indicate more complexity. However, Kolmogorov did not address the method to find the shortest description of a given string of characters. Therefore, the complexity is most commonly approximated by general compression algorithms (e.g., as implemented in zip or gzip programs) where the length of a compressed sequence gives an estimate of its complexity (i.e., a more complex string will be less compressible) [60]. The calculation of a distance between sequences using complexity (compression) is relatively straightforward (Fig. 2). This procedure takes the sequences being compared (*x* = ATGTGTG and *y* = CATGTG) and concatenates them to create one longer sequence (*xy* = ATGTGTGCATGTG). If *x* and *y* are exactly the same, then the complexity (compressed length) of *xy* will be very close to the complexity of the individual *x* or *y*. However, if *x* and *y* are dissimilar, then the complexity of *xy* (length of compressed *xy*) will tend to the cumulative complexities of *x* and *y*. Of course, there are as many different information-based distances as there are methods to calculate complexity. For example, Lempel–Ziv complexity [61] is a popular measure that calculates the number of different subsequences encountered when viewing the sequence from beginning to end (Fig. 2). Once the complexities of the sequences are calculated, a measure of their differences (e.g., the normalized compression distance [62]) can be easily computed. Many DNA-specific compression algorithms are currently being applied to new types of problems [63].

Another example of an information measurement often applied to biological sequences is entropy. This measurement is not similar to the entropy referenced in thermodynamics. Reportedly, Claude Shannon, who was a mathematician working at Bell Labs, asked John von Neumann what he should call his newly developed measure of information content; "Why don't you call it entropy," said von Neumann, "[…] no one understands entropy very well so in any discussion you will be in a position of advantage […]" [64]. The concept of Shannon entropy came from the observation that some English words, such as "the" or "a", are very frequent and thus unsurprising. Thus, these words are redundant because the message can probably be understood without them. The real essence of the message comes from words that are rare, such as "treasure" or "elixir". Therefore, Shannon developed a formula to quantify the uncertainty (entropy) of finding a given element (word) in an analyzed sequence (text). Using Shannon's concept, Kullback and Leibler [65] introduced a relative entropy measure (Kullback–Leibler divergence, KL) that allowed for a comparison of two sequences. The procedure involves the calculation of the frequencies of symbols or words in a sequence and the summation of their entropies in the compared sequences (Additional file 1: Figure S1).

Both information-theory concepts (complexity and entropy) have a clear association despite their methodical differences. For instance, a low-complexity sequence (e.g., AAAAAAAAA) will have smaller entropy than a more complex sequence (e.g., ACCTGATGT). The application of information theory in the field of sequence analysis and comparison has exploded in recent years, ranging from global (block entropies and coverage) to local genome analyses (transcription factor binding sites, sequences as time-series and entropic profiles) [39]. Additionally, retrieving higher-level correlations in gene mapping and protein–protein interaction networks and the striking resemblance with communication systems is attracting research attention to this field.

## How are alignment-free methods used in next-generation sequencing data analysis?

The data volume of samples sequenced so far (estimated to be only 10^−20^% of the total DNA on Earth [66]) is already challenging the storage and processing capacities of modern computers. In particular, the amount of data generated via next-generation sequencing is swiftly outpacing analytics capabilities, mainly due to the computationally intensive multiple alignment step. Alignment-free methods not only provide a significant increase in speed over primary next-generation sequencing applications (e.g., expression profiling [67–70], genetic variant calling [71–74], de novo genome assembly [75–77], phylogenetic reconstruction [78–81], and taxonomic classification in metagenomic studies [82–86]) (Table 1), but also offer ways to obtain biologically meaningful information directly from raw next-generation sequencing data.

**Table 1:** Alignment-free sequence comparison tools available for next-generation sequencing data analysis

Category	Analysis	Tool	Primary features	Implementation	Reference	URL
Mapping	Transcript quantification	kallisto	Transcript abundance quantification from RNA-seq data (uses pseudoalignment for rapid determination of read compatibility with targets)	Software (C++)	[69]	https://pachterlab.github.io/kallisto/
		Sailfish	Estimation of isoform abundances from reference sequences and RNA-seq data (*k*-mer based)	Software (C++)	[67]	http://www.cs.cmu.edu/~ckingsf/software/sailfish/
		Salmon	Quantification of the expression of transcripts using RNA-seq data (uses *k*-mers)		[70]	https://combine-lab.github.io/salmon/
		RNA-Skim	RNA-seq quantification at transcript-level (partitions the transcriptome into disjoint transcript clusters; uses *sig*-mers, a special type of *k*-mers)	Software (C++)	[68]	http://www.csbio.unc.edu/rs/
	Variant calling	ChimeRScope	Fusion transcript prediction using gene *k*-mers profiles of the RNA-seq paired-end reads	Software (Java)	[74]	https://github.com/ChimeRScope/ChimeRScope/wiki
		FastGT	Genotyping of known SNV/SNP variants directly from raw NGS sequence reads by counting unique *k*-mers	Software (C)	[73]	https://github.com/bioinfo-ut/GenomeTester4/
		Phy-Mer	Reference-independent mitochondrial haplogroup classifier from NGS data (*k*-mer based)	Software (Python)	[157]	https://github.com/danielnavarrogomez/phy-mer
		LAVA	Genotyping of known SNPs (dbSNP and Affymetrix's Genome-Wide Human SNP Array) from raw NGS reads (*k*-mer based)	Software (C)	[71]	http://lava.csail.mit.edu/
		MICADo	Detection of mutations in targeted third-generation NGS data (can distinguish patients’ specific mutations; algorithm uses *k*-mers and is based on colored de Bruijn graphs)	Software (Python)	[72]	http://github.com/cbib/MICADo
	General mapper	Minimap	Lightweight and fast read mapper and read overlap detector (uses the concept of “minimazers”, a special type of *k*-mers)	Software (C)	[77]	https://github.com/lh3/minimap
Assembly	De novo genome assembly	MHAP	Produces highly continuous assembly (fully resolved chromosome arms) from third-generation long and noisy reads (10 kbp) using a dimensionality reduction technique MinHash	Software (Java)	[76]	https://github.com/marbl/MHAP
		Miniasm	Assembler of long noisy reads (SMRT, ONT) using the Overlap-Layout Consensus (OLC) approach without the necessity of an error correction stage (uses minimap)	Software (C)	[77]	https://github.com/lh3/miniasm
		LINKS	Scaffolding genome assembly with error-containing long sequence (e.g., ONT or PacBio reads, draft genomes)	Software (Perl)	[75]	https://github.com/warrenlr/LINKS/
	Read clustering	afcluster	Clustering of reads from different genes and different species based on *k*-mer counts	Software (C++)	[158]	https://github.com/luscinius/afcluster
		QCluster	Clustering of reads with alignment-free measures (*k*-mer based) and quality values	Software (C++)	[159]	http://www.dei.unipd.it/~ciompin/main/qcluster.html
	Reads error correction	Lighter	Correction of sequencing errors in raw, whole genome sequencing reads (*k*-mer based)	Software (C++)	[94]	https://github.com/mourisl/Lighter
		QuorUM	Error corrector for Illumina reads using k-mers	Software (C++)	[93]	https://github.com/gmarcais/Quorum
		Trowel		Software (C++)	[95]	https://sourceforge.net/projects/trowel-ec/
Metagenomics	Assembly-free phylogenomics	AAF	Phylogeny reconstruction directly from unassembled raw sequence data from whole genome sequencing projects; provides bootstrap support to assess uncertainty in the tree topology (*k*-mer based)	Software (Python)	[78]	https://github.com/fanhuan/AAF
		kSNP v3	Reference-free SNP identification and estimation of phylogenetic trees using SNPs (based on *k*-mer analysis)	Software (C)	[80, 81]	https://sourceforge.net/projects/ksnp/files/
		NGS-MC	Phylogeny of species based on NGS reads using alignment-free sequence dissimilarity measures d_2_* and d_2_^S^ under different Markov chain models (using *k*-words)	R package	[79, 160]	http://www-rcf.usc.edu/~fsun/Programs/NGS-MC/NGS-MC.html
	Species identification/taxonomic profiling	CLARK	Taxonomic classification of metagenomic reads to known bacterial genomes using *k*-mer search and LCA assignment	Software (C++)	[84]	http://clark.cs.ucr.edu/
		FOCUS	Reports organisms present in metagenomic samples and profiles their abundances (uses composition-based approach and non-negative least squares for prediction)	Web service Software (Python)	[161]	http://edwards.sdsu.edu/FOCUS/
		GSM	Estimation of abundances of microbial genomes in metagenomic samples (*k*-mer based)	Software (Go)	[162]	https://github.com/pdtrang/GSM
		Mash	Species identification using assembled or unassembled Illumina, PacBio, and ONT data (based on MinHash dimensionality-reduction technique)	Software (C++)	[163]	https://github.com/marbl/mash
		Kraken	Taxonomic assignment in metagenome analysis by exact *k*-mer search; LCA assignment of short reads based on a comprehensive sequence database	Software (C++)	[83]	https://ccb.jhu.edu/software/kraken/
		LMAT	Assignment of taxonomic labels to reads by *k*-mers searches in precomputed database	Software (C++/Python)	[82]	https://sourceforge.net/projects/lmat/
		stringMLST	*k*-mer-based tool for MLST directly from the genome sequencing reads	Software (Python)	[86]	http://jordan.biology.gatech.edu/page/software/stringMLST
		Taxonomer	*k*-mer-based ultrafast metagenomics tool for assigning taxonomy to sequencing reads from clinical and environmental samples	Web service	[164]	http://taxonomer.iobio.io/
	Other	d2-tools	Word-based (*k*-tuple) comparison (pairwise dissimilarity matrix using d2S measure) of metatranscriptomic samples from NGS reads	Software (Python/R)	[56, 165]	https://code.google.com/p/d2-tools/
		VirHostMatcher	Prediction of hosts from metagenomic viral sequences based on ONF using various distance measures (e.g., d_2_)	Software (C++)	[153]	https://github.com/jessieren/VirHostMatcher
		MetaFast	Statistics calculation of metagenome sequences and the distances between them based on assembly using de Bruijn graphs and Bray–Curtis dissimilarity measure	Software (Java)	[166]	https://github.com/ctlab/metafast

The up-to-date list of currently available programs can be found at http://www.combio.pl/alfree/tools/. Accessed 23 August 2017

*LCA* lowest common ancestor, *NGS* next-generation sequencing, *SNP* single-nucleotide polymorphism, *SNV* single-nucleotide variant

For example, alignment-free tools for transcript quantification (Kallisto [69], Sailfish [67], Salmon [70]) show that most of the information provided by aligners is not necessary for high-quality estimation of transcript levels. These tools build an index of *k*-mers from a reference set of transcripts and then calculate the expression by matching them to each sequencing read directly. Such “pseudoalignment” [69] describes the relationship between a read and a set of compatible transcripts. Grouping pseudoalignments belonging to the same set of transcripts allows one to directly infer the expression of each transcript model. This approach to quantify gene/transcript expression levels from RNA sequencing reads is both 10–100 times faster than any of the alignment-based methods and at least as accurate as best-performing alignment-based workflows (e.g., TopHat-Cufflinks) [87, 88].

Another major application of next-generation sequencing technologies includes profiling of genomic variabilities, such as single nucleotide/variant polymorphisms. These genomic alterations are typically detected by genotype calling on mapped reads (e.g., Samtools mpileup [89] and GATK HaplotypeCaller [90]). However, alignment-free tools (FastGT [73] and LAVA [71]) allow for genotyping of known variants directly from next-generation sequencing data, based on *k*-mer analysis. Since these methods are 1–2 orders of magnitude faster than traditional mapping-based detection, they seem to be ideally suited for clinical applications, where sequencing data from a large number of individuals need to be processed in a timely manner. For example, MICADo analyzes third-generation sequencing reads for each patient sample within the context of the data of the whole cohort in order to capture patient-specific mutations [72] and ChimeRScope predicts fusion transcripts with potential oncogenic functions, based on the *k*-mer profiles of the RNA-seq paired-end reads [74].

Conventional next-generation sequencing computation came of age with the emergence of the MapReduce functional pattern to orchestrate parallelization of order-free operations [90]. It is, therefore, of no surprise that it would be advantageous to implement alignment-free methods for the same pattern. Such a solution comes naturally to the word counting implementation of *k*-mer analysis and may have further reaching implications for the molecular applications discussed in the previous paragraph, and it is also found to be a natural fit to scale-free approaches to alignment-free methods [91]. This solution was successfully put to the test in the simultaneous screening of 20 *Streptococcus pneumoniae* genomes for shared suffixes in a volunteer distributed computing implementation of that alignment-free MapReduced implementation [92].

One of the most demanding tasks in today's biology includes assembly of the newly sequenced genomes. In standard applications, it requires an error correction step and construction of the genome scaffold based on read similarity (sequence overlaps). Several alignment-free tools have been created to correct sequencing reads (e.g., Quorum [93]), designed mainly to be fast and memory efficient (e.g., Lighter [94] using sampling of *k*-mers instead of counting), as well as highly accurate (e.g., Trowel [95] using quality threshold rather than coverage cut-off in order to extract trusted *k*-mers).

The advent of third-generation sequencing technologies (PacBio and Oxford Nanopore) provides an opportunity to study new genomes with unprecedented speed and quality. However, the noisy nature of sequencing data demands dedicated solutions to access more complex genomes. The MinHash Alignment Process was designed for this task employing probabilistic, locality-sensitive hashing. Integration of the MinHash Alignment Process with the Celera Assembler enabled reference-grade de novo assemblies of several eukaryotic genomes [76]. Another example includes currently developed Miniasm de novo assembler [77], which uses an overlap-layout-consensus approach [96]. Miniasm requires all-versus-all read self-mappings as input, which can be obtained by the alignment-free Minimap tool. Finally, LINKS [75] is a genomic tool designed for scaffolding genome assemblies with long reads (including draft genomes). The major advantage of this method is the use of paired *k*-mers from variable long sequence sources without a need of read correction.

Metagenomics, the study of genomic sequences obtained directly from the environment (e.g., aquatic ecosystems, human body), has become a primary application of alignment-free methods, in particular programs designated for fast and precise profiling of microbial communities. For example, Kraken [83] and CLARK [84] are top-performing tools designed for this task—they assign taxonomic labels to individual reads in large datasets with near perfect accuracy (precision > 99%), even in the presence of unknown organisms. These programs perform metagenomic classification of next-generation sequencing reads based on the analysis of shared *k*-mers between an input read and each genome from a precomputed database. Kraken additionally assigns each *k*-mer to the lowest common ancestor of all organisms whose genomes contain corresponding *k*-mers (Additional file 1: Figure S2). The evaluation of the accuracy and speed of 14 widely used metagenome analysis tools [97] showed that Kraken and CLARK are top state-of-the-art tools with the highest speed, accuracy, and sensitivity (i.e., the fraction of reads that is correctly classified).

The alignment-free techniques are continuously being applied to new next-generation sequencing based solutions, for example, phylogenomics (reviewed in [57]), where advances have facilitated construction of high-quality phylogenies directly from raw, unassembled genome sequence data, bypassing both genome assembly and alignment. Assembly and alignment-free phylogenetic tools are already available on the market (AAF [78], NGS-MC [79], and kSNP [80, 81]) and although algorithmically different (e.g., based on single-nucleotide polymorphism calls or various dissimilarity measures), all of them are capable of phylogeny reconstruction of non-model species even in cases of low sequence coverage or lack of a reference genome. In addition, the AAF program provides bootstrap support to assess the confidence of tree topology and addresses problems of homoplasy, sequencing error, and incomplete coverage.

## Where else can alignment-free sequence comparison methods be applied?

Progress over the past two decades has led alignment-free research from bioinformatics “curiosities” to a broadening range of successful applications that accompany mainstream biology [37].

Distantly related, remote sequences that evolve beyond recognizable similarity are one of the most classic applications of alignment-free mastering. For example, alignment-free approaches were successfully employed in functional annotation of unknown G-protein-coupled receptor (integral cell membrane proteins that play a key role in transducing extracellular signals and have great relevance for pharmacology) sequences that could not be assigned to any previously known receptor family [98]. Another rising trend for the use of word-based alignment-free methods is the detection of functional and/or evolutionary similarities among regulatory sequences (e.g., promoters, enhancers, and silencers) to estimate their in vivo activities in different organisms (flies and mammals, including humans) [99–103].

Sequence rearrangements are particularly well handled by alignment-free sequence analyses*.* Recent studies described the mosaic structure of viral and bacterial genomes (e.g., by characterizing the recombination break points in HIV-1 strain and *Escherichia coli* genomes). This analysis provides new evidence for the long-held suspicion that animal *E. coli* pathogens can also infect humans [104]. Another study [105] discovered a clear signal for a pair of *E. coli* genomes that had undergone an engineered 125-kb horizontal gene transfer 20 years ago. Alignment-free measures were also applied to detect domain shuffling signatures in proteins [106] and to identify the members of complex multidomain proteins, such as kinases [107].

Horizontal gene transfer strongly complicates the task of reconstructing the evolutionary history of genes and species, and alignment-free methods have also proved to be helpful in this field. For example, in a comprehensive study of bacterial genomes, the authors used oligonucleotides as genomic signatures and showed that horizontal gene transfers accounted for 6% of the genomes on average [108]. Furthermore, the statistical relationships between genomic signatures among several thousand species provided information about possible donor taxa for the identified foreign sequences. In other studies [109, 110], alignment-free approaches were applied to the genomes of the human pathogen *Staphylococcus aureus* and recovered regions of lateral origin that corresponded to genes involved in transport, antibiotic resistance, pathogenicity, and virulence.

Whole-genome phylogeny [111] is another area where alignment-free methods play an increasing role. Many studies [34, 112–118] addressed the phylogenetic reconstruction of prokaryotes, such as the whole-genome phylogeny of *E. coli* O104:H4, which was the strain that caused the 2011 outbreak in Germany. The analysis revealed a direct line of ancestry leading from a putative typical enteroaggregative *E. coli* ancestor through the 2001 strain to the 2011 outbreak strain [113]. The alignment-free based phylogeny of almost a hundred Zika virus strains suggested that this mosquito-borne flavivirus originated from Africa and then spread to Asia, the Pacific islands, and throughout the Americas [119]. Alignment-free methods have recently been applied to infer phylogenetic relationships among eukaryotic species (fungi [120], plants [121], and mammals [35]); the resulting trees were extremely similar to the species trees created by the manually curated NCBI taxonomic database, which reflects the current taxonomic consensus in the literature.

Sequence classification is another field that might benefit from bringing together different alignment-free approaches, such as grouping expressed sequences tags that originate from the same locus or gene family [122], clustering expressed sequence tag sequences with full-length cDNA data [123], and aggregating gene and protein sequences into functional families [124–126]. Alignment-free methods are also used to recognize and classify antigens that are encoded in a sequence in a subtle and recondite manner that is not identifiable by sequence alignment. A recent approach [127, 128] based on the statistical transformation of protein sequences into uniform vectors with various amino acid properties showed an impressive prediction accuracy of up to 89% in discriminating positive and negative sets of bacterial, viral, and tumor antigen datasets. Another common use of alignment-free methods is the classification of species based on a short DNA sequence fragments that can act as true taxon barcodes [129–133].

The available alignment-free-based software for general sequence comparison are listed in Table 2. For convenience, we categorized the listed programs into basic research tasks, such as small scale pairwise/multiple sequence comparisons, whole genome phylogeny (from viral to mammalian scale), BLAST-like sequence similarity search, identification of horizontally transferred genes and recombination events, as well as annotation of long non-coding RNAs and regulatory elements.

**Table 2:** Alignment-free sequence comparison tools available for research purposes

Category	Name	Features	Implementation	Reference	URL
Pairwise and multiple sequence comparison	ALF	Calculation of pairwise similarity scores (using N2 measure) for sequences in fasta file	Software (C++)	[101]	https://github.com/seqan/seqan/tree/master/apps/alf
	Alfree	25 word-based measures, 8 IT-based measures, 3 graph-based measures, W-metric	Web service Software (Python)	This article	http://www.combio.pl/alfree
	decaf + py	13 word-based measures, Lempel–Ziv complexity-based measure, average common substring distance, W-metric	Software (Python)	[52, 53]	http://bioinformatics.org.au/tools/decaf+py/
	multiAlignFree	Multiple alignment-free sequence comparison using five word-based statistics	R package	[167]	http://www-rcf.usc.edu/~fsun/Programs/multiAlignFree/
	NASC	Non-aligned sequence comparison: four word-based measures and 2 IT-based measures	Matlab framework	[38]	http://web.ist.utl.pt/susanavinga/NASC/
Whole-genome phylogeny	ALFRED ALFRED-G	Phylogenetic tree reconstruction based on the average common substring approach	Software (C++)	[168, 169]	http://alurulab.cc.gatech.edu/phylo
	andi	Computation of evolutionary distances between closely related genomes by approximation of local alignments (*k*-mer based d_a_ measure); scalable to thousands of bacterial genomes	Software (C)	[170]	https://github.com/evolbioinf/andi/
	CAFE	Alignment-free analysis platform for studying the relationships among genomes and metagenomes (offers 28 word-based dissimilarity measures)	Software (C)	[171]	https://github.com/younglululu/CAFE
	CVTree3	Phylogeny reconstruction from whole genome sequences based on word composition	Web service	[172, 173]	http://tlife.fudan.edu.cn/cvtree3
	DLTree	Automated whole genome/proteome-based phylogenetic analysis based on alignment-free dynamical language method	Web Service	[174]	http://dltree.xtu.edu.cn
	FFP	Feature frequency profile-based measures for whole genome/proteome comparisons (from viral to mammalian scale)	Software (C/Perl)	[34, 55, 112]	https://sourceforge.net/projects/ffp-phylogeny/
	jD2Stat (JIWA)	Generation of the distance matrix using *D*_*2*_ statistics to extract *k*-mers from large-scale unaligned genome sequences	Software (Java)	[54]	http://bioinformatics.org.au/tools/jD2Stat/
	kr	Efficient word-based estimation of mutation distances from unaligned genomes	Software (C)	[175]	http://guanine.evolbio.mpg.de/cgi-bin/kr2/kr.cgi.pl
	FSWM/kmacs/Spaced	Three tools for alignment-free sequence comparison based on inexact word matches	Software (C++) Web service	[36, 176]	Software currently unavailable Software currently unavailable Software currently unavailable
	SlopeTree	Whole genome phylogeny that corrects for HGT	Software (C++)		http://prodata.swmed.edu/download/pub/slopetree_v1/
	Underlying Approach	Phylogeny of whole genomes using composition of subwords	Software (Java)	[139]	http://www.dei.unipd.it/~ciompin/main/underlying.html
Sequence similarity search tool	RAFTS3	Searches of similar protein sequences against a protein database (>300 times faster than BLAST)	Matlab	[177]	https://sourceforge.net/projects/rafts3/
Annotation of long non-coding RNA	FEELnc	Prediction of lncRNAs from RNA-seq samples based word frequencies and relaxed open reading frames	Software (Perl/R)	[178]	https://github.com/tderrien/FEELnc
	lncScore	Identification of long non-coding RNA from assembled novel transcripts	Software (Python)	[152]	https://github.com/WGLab/lncScore
Horizontal gene transfer	alfy	Alignment-free local homology calculation for detecting horizontal gene transfer	Software (C)	[104, 109]	http://guanine.evolbio.mpg.de/alfy/
	rush	Detection of recombination between two unaligned DNA sequences	Software (C)	[105]	http://guanine.evolbio.mpg.de/rush/
	Smash	Identification and visualization of DNA rearrangements between pairs of sequences	Software (C)	[179]	http://bioinformatics.ua.pt/software/smash/
	TF-IDF	Detection of HGT regions and the transfer direction in nucleotide/protein sequences	Software (C++)	[110, 180]	https://github.com/congyingnan/TF-IDF
Regulatory elements	D2Z	Identification of functionally related homologous regulatory elements	Software (Perl)	[102]	http://veda.cs.uiuc.edu/d2z/
	MatrixREDUCE	Prediction of functional regulatory targets of TFs by predicting the total affinity of each promoter and orthologous promoters	Software (Python)	[181]	https://systemsbiology.columbia.edu/matrixreduce
	RRS	Detection of functionally similar group of enhancers and their regions	Software (Perl/C)	[182]	http://goo.gl/7gW578
Sequence clustering	d2_cluster	Word-based clustering EST and full-length cDNA sequences	Software (C)	[123]	https://github.com/shaze/wcdest/
	d2-vlmc	Word-based clustering of metatranscriptomic samples using variable length Markov chains	Software (Python)	[183]	https://d2vlmc.codeplex.com/
	mBKM	Clustering of DNA sequences using Shannon entropy and Euclidean distance	Software (Java)	[124]	https://github.com/Huiyang520/DMk-BKmeans
	kClust	Large-scale clustering of protein sequences (down to 20–30% sequence identity)	Software (C++)	[125]	https://github.com/soedinglab/kClust
Other	COMET	Rapid classification of HIV-1 nucleotide sequences into subtypes based on prediction by partial matching compression	Web service	[184]	https://comet.lih.lu/
	PPI	Identification of protein–protein interaction by coevolution analysis using discrete Fourier transform	Software (Python)	[185]	https://github.com/cyinbox/PPI
	VaxiJen	Antigen prediction based on uniform vectors of principal amino acid properties	Web service	[127]	http://www.ddg-pharmfac.net/vaxijen/VaxiJen/VaxiJen.html

The up-to-date list of currently available programs can be found at http://www.combio.pl/alfree/tools/. Accessed 23 August 2017

*HGT* horizontal gene transfer, *IT* information theory

## How to use alignment-free methods for research purposes

Among programs listed in Table 2, CAFE is an example of a general purpose alignment-free software that allows exploration of relationships among multiple DNA sequences through a graphical user interface. The tool integrates 28 dissimilarity measures based on *k*-mer analysis, including ten conventional (e.g., Euclidean, Manhattan, d_2_), 15 based on presence/absence of *k*-mers (e.g., Jaccard and Hamming distances) and three state-of-the-art measures based on background adjusted *k*-mer counts (i.e., CVTree, d^*^_2_ and *d* ^*S*^_*2*_). The resulting pairwise dissimilarities among the sequences form a distance matrix, which can be directly saved in a standard PHYLIP format. In addition, CAFE presents pairwise dissimilarity measures in a form of different visualizations, including dendrogram (i.e., tree illustrating the clustering of the sequences), heatmap, principal coordinate analysis, and network display.

**Fig. 3:**
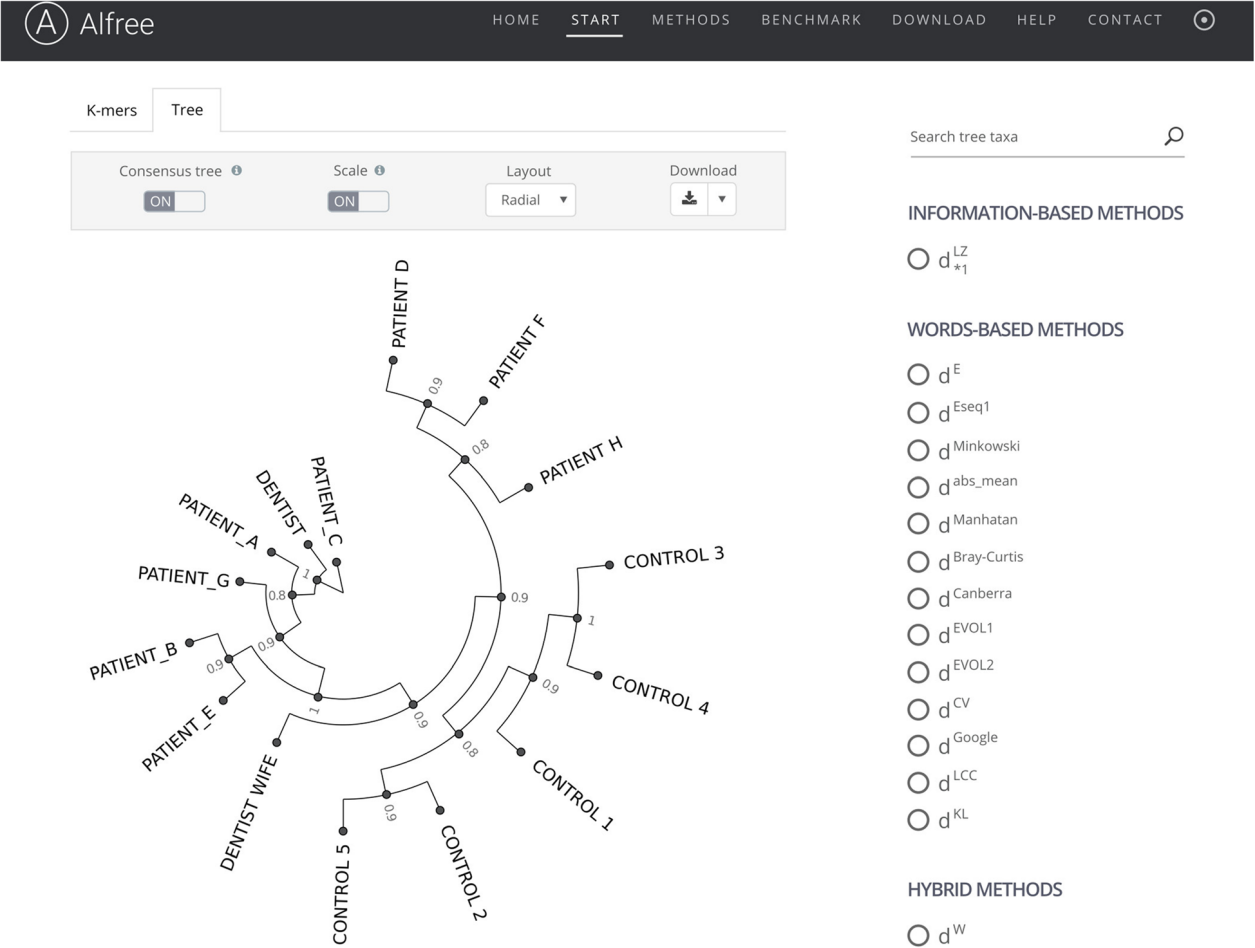
Snapshot of the results returned by the alignment-free web tool (Alfree) for “example 1”: HIV viral sequences obtained from dental patients in Florida [186]. Briefly, in the late 1980s some patients of an HIV-positive dentist in Florida were diagnosed as infected with HIV. An investigation by the Centers for Disease Control and Prevention did not uncover any hygiene lapses that could result in infection of patients. However, sequence comparison of the gene encoding gpg120 isolated from HIV strains from the dentist, his patients, and other individuals revealed that *PATIENT_A*, *PATIENT_B*, *PATIENT_C*, *PATIENT_E*, and *PATIENT_G* became infected while receiving dental care [183]. The phylogeny shown is based on the gp120 viral protein sequences from the dentist, the dentist’s wife (*DENTIST WIFE*), eight patients (*PATIENT_A* to *PATIENT H*), and five individuals that never had contact with the accused (*CONTROL 1*, *2*, *3*, *4*, and *5*). The sphylogram was obtained as a majority-rule consensus tree that summarizes the agreement across 15 alignment-free methods (support values in scale from 0 to 1 are shown for every node of the tree). The web interface of the Alfree portal also provides an example case of phylogenetic reconstruction of mitochondrial genomes of 12 primates. Several additional options are available to explore and visualize the sequence comparison results, including selection of individual method, re-rooting trees, changing tree layouts, as well as collapsing or expanding different parts of the tree

Most of the listed tools, including CAFE, are stand-alone programs (only a few were implemented as web services) and therefore may require some specific installation procedures. In this summary article, we have launched a novel, publicly accessible web application for alignment-free sequence comparisons/phylogeny, in a way that anyone can give it a try without any programming deployment effort (no expertise required). The web application (http://www.combio.pl/alfree) uses 38 popular alignment-free methods to calculate distances among given nucleotide or protein sequences. By default, running an analysis is a “one-step process”—after providing the input sequences the server will execute the alignment-free analysis in a fully automated mode without the need for further user intervention. The results are reported as a consensus phylogenetic tree that summarizes the agreement between various individual methods’ trees, thus allowing users to assess the reliability of given phylogenetic relationships across different methods (Fig. 3). Users can also browse trees obtained by individual methods as well as inspect distance measures for any pair of query sequences by using interactive heat maps and tables.

## How well do alignment-free methods work?

The performance of alignment-free methods has improved greatly since the introduction of the first alignment-free measure exactly 30 years ago [134]. The challenge today, however, is not a lack of alignment-free algorithms (there are almost 100 published methods), but the number of benchmarking approaches to alignment-free sequence comparison—once a new method is published, a new evaluation procedure and/or selected dataset is also introduced. For example, the majority of algorithms have been evaluated using various sets of simulated DNA sequences [54, 135, 136], primate/mammalian mitochondrial genomes [40, 61, 137, 138], whole prokaryotic genomes/proteomes [117, 139], selected plant genomes [121, 140], small subsets of homologous genes [141, 142], and different combinations thereof [36, 139].

Giving the heterogeneity of testing procedures, it has been quite an achievement by four independent studies to evaluate several classic distance measures for their application under different scenarios of sequence evolution. The first benchmark, by Vinga and Almeida (2004) [143], compared the accuracy of six word-based methods in recognition of structurally and evolutionary relationships among proteins. Höhl and Ragan [52, 53] tested the accuracy of nine alignment-free methods in the construction of phylogenetic trees using homologous proteins representing a wide range of phylogenetic distances. Both research groups showed that, in general, tested alignment-free methods can be as good as alignment algorithms and, as reported in [52], may perform even better in case of protein sequences that underwent domain shuffling events. Dai and colleagues (2008) [99] confronted nine alignment-free distance measures and two alignment-based approaches (Needleman–Wunsch and Smith–Waterman alignment methods) in annotation of functionally related regulatory sequences in human and fly. Almost all tested alignment-free methods detected statistically relevant similarities in sequence compositions in contrast to alignment-based methods that showed only limited correspondence recognizable by alignments. In a recent benchmark, Bernard and colleagues (2016 [33]) used simulated and empirical microbial genomes to test the sensitivity of nine alignment-free methods under different evolutionary schemes. All approaches generated biologically meaningful phylogenies—alignment-free methods were most sensitive to the extent of sequence divergence, less sensitive to low and moderate frequencies of horizontal gene transfer, and most robust against genome rearrangements.

We extended the benchmark of Vinga and Almeida (2004) to test 33 popular alignment-free methods (as well as the Smith–Waterman algorithm—the most accurate algorithm for sequence alignments) in the classification of structural and evolutionary relationships between protein sequences from the SCOPe/ASTRAL database [144]. This resource provides a high-quality structural classification of proteins at four levels: class, folds, superfamilies, and families (for details see Additional file 2: Table S1). As in the previous study [143], we used a representative subset of the SCOPe database (containing proteins sharing less than 40% identity) as a reference to test 25 word-based and eight information theory-based alignment-free methods along with different combinations of their input parameters, such as word size (from 1 to 4) and vector type (e.g., counts, frequencies, etc.). The performance of each method was assessed using AUC statistics (area under the receiver operating curve; for details about methods see Additional file 3: Supplementary methods).

The alignment-based algorithm (Smith–Waterman algorithm) was outperformed at all SCOP levels—i.e., class, class fold, superfamily, family (AUCs 0.62, 0.67, 0.78, 0.81)—by two word-based measures: normalized Google distance [59] (AUCs 0.63, 0.78, 0.80, 0.84) and Bray–Curtis distance [145] (AUCs 0.63, 0.77, 0.80, 0.84) (Additional file 2: Table S1). Three other word-based methods, including two variants of Squared Euclidean distance [53] as well as the Canberra distance [146], though less accurate in recognition of relationships within class, obtained higher overall scores (AUCs 0.744, 0.733, and 0.725, respectively) than the Smith–Waterman algorithm (AUC 0.72). These results support the assumption—very often taken for granted—that alignment-free methods can produce more accurate results than alignment-based solutions when applied to homologous sequences of low similarity. Interestingly, the Smith–Waterman algorithm was outperformed only by word-based methods with short *k*-mers of one to two residues, indicating that the conservation and order of longer sequence stretches are generally not preserved in the sequences, and the relationship between alignment similarity score and structural/evolutionary relationship breaks down. As alignment-free methods do not depend on where the words are found in the sequence, they are typically not confused by the complexities caused by mismatches, gaps, and sequence inversions that are often found in this type of distantly related homolog. It is also interesting to note that word-based methods achieved higher accuracy (AUC 0.67 ± 0.04) than information-theory based solutions (AUC 0.61 ± 0.06) (Additional file 2: Table S1). Although explanation of this fact is not straightforward, it may indicate that compression procedures included in currently selected methods do not decipher the complexity of highly variable protein sequence, which would explain the broader application of the information-theory based methods in DNA sequence analyses. The full results of the benchmark can be interactively explored [145].

Remarkably, the duration time for the calculation of approximately 22 million pairwise protein comparisons by the Smith–Waterman algorithm took exactly 3 days, which was more than 1000-fold slower than the alignment-free methods (Additional file 3: Supplementary methods). On average, these methods need 4 minutes to complete the task, and the fastest approach (Hamming distance [146]) ran the analysis in only 19 seconds.

The implementations of all alignment-free methods used in this study are provided as a stand-alone Python application [147]. We also supplement this article with the benchmark dataset [148] for reference analysis that can be readily reproduced by enthusiasts or developers building new alignment-free solutions.

## Conclusions

As sequencing technology becomes less expensive and more ubiquitous, the computational challenges of sequence analyses will become even more prominent. This issue pushes the current focus of development towards faster alignment-independent solutions. Will these new techniques spell doom for traditional alignments? Most likely not in the authors’ lifetime. Alignment is still irreplaceable in many aspects of today's biology, such as the annotation of conserved protein domains and motifs, tracking phenotype-related sequence polymorphisms, reconstruction of ancestral DNA sequences, determining the rate of sequence evolution, and homology-based modeling of three-dimensional protein structures. In addition, the research on, and the development of, alignment-free methods is still relatively young, holding considerable potential for improvement, whereas alignment approaches are already mature and only a few alignment-free methods have really challenged the validity and reliability of alignment-based techniques.

Most published articles about alignment-free sequence comparison methods are still mainly technical, exploring their mathematical foundations and theoretical performance (versus alignment-based approaches), very often evaluated with individually selected, mostly simulated, data sets. Although many alignment-free programs exist (as shown in Tables 1 and 2), the majority of published alignment-free methods are still not supplemented with software implementations and thus cannot easily be compared on common sets of data. The absence of well-defined benchmarks covering various evolutionary scenarios of sequence divergence creates a major obstacle for researchers who simply need to know the current “best” tool. Consequently, it is still difficult to state which alignment-free method might be particularly suited for a certain task. The stage thus appears to be now set for application of alignment-free methods on real world data sets, which seems to be the only way for these methods to be widely accepted by scientists in biology and related fields [149].

Although alignment-free methods are computationally relatively easily scalable to multigenome data, they do have some “skeletons in their closet”. For example, using long *k*-mers in word-based methods may impose a substantial memory overhead (the total number of possible DNA words of length 14 is 4^14^, which is about 4 GB). Although information-theory methods that are based on the compression algorithms are more memory efficient and computationally inexpensive, they may fail to decipher complex organization levels in the sequences [39] (also shown in results obtained in this study; Additional file 2: Table S1). Some of these issues have already been addressed; for example, recent reports demonstrate the reasonable memory usage of word-based approaches (with long 25-mers) for phylogenetic reconstruction of more than 100 bacterial genomes [54, 150].

Nevertheless, alignment-free algorithms are rapidly extending the range of their applications [151–154] and answering previously intractable questions in phylogenomics and horizontal gene transfer (reviewed in [57]), population genetics (reviewed in [111]), evolution of regulatory sequences, and links between the genome and epigenome (reviewed in [155]). Disadvantages of next-generation sequencing data processing and analysis seem to be particularly well addressed by the alignment-free methods (reviewed in [156]). The currently dominant *k*-mer approaches are bound to novel measures for biological applications (e.g., Google distance [59]) and application of advanced information theory-based methods should improve the available alignment-free and alignment-based tool box. From this fair competition between alignment-based and alignment-free camps, scientists can get only the best. In this respect, the next years should be very exciting.

## Additional files

Additional file 1: **Figures S1.** and **Figure S2.** Kraken algorithm for taxonomic labeling of metagenomic DNA sequences (based on Wood and Salzberg, 2014) [83]. (DOCX 864 kb)

Additional file 2: Table S1. Ranking list of alignment-free methods and the Smith-Waterman algorithm based on the area under the curve measures across four structural levels of the SCOP2 database. (DOCX 39 kb)

Additional file 3: Supplementary methods. (DOCX 37 kb)
