# Supplementary material for: Alignment-free sequence comparison: benefits, applications, and tools
Source: Genome Biol. 2017 Oct 3;18:186. doi: 10.1186/s13059-017-1319-7 (PMC5627421; doi:10.1186/s13059-017-1319-7)
Supplement: Supplementary file 1 — Figures S1. and Figure S2. Kraken algorithm for taxonomic labeling of metagenomic DNA sequences (based on Wood and Salzberg, 2014) [83]. (DOCX 864 kb) [file 13059_2017_1319_MOESM1_ESM.docx]

Additional file 1

**An introduction to alignment-free sequence comparison**


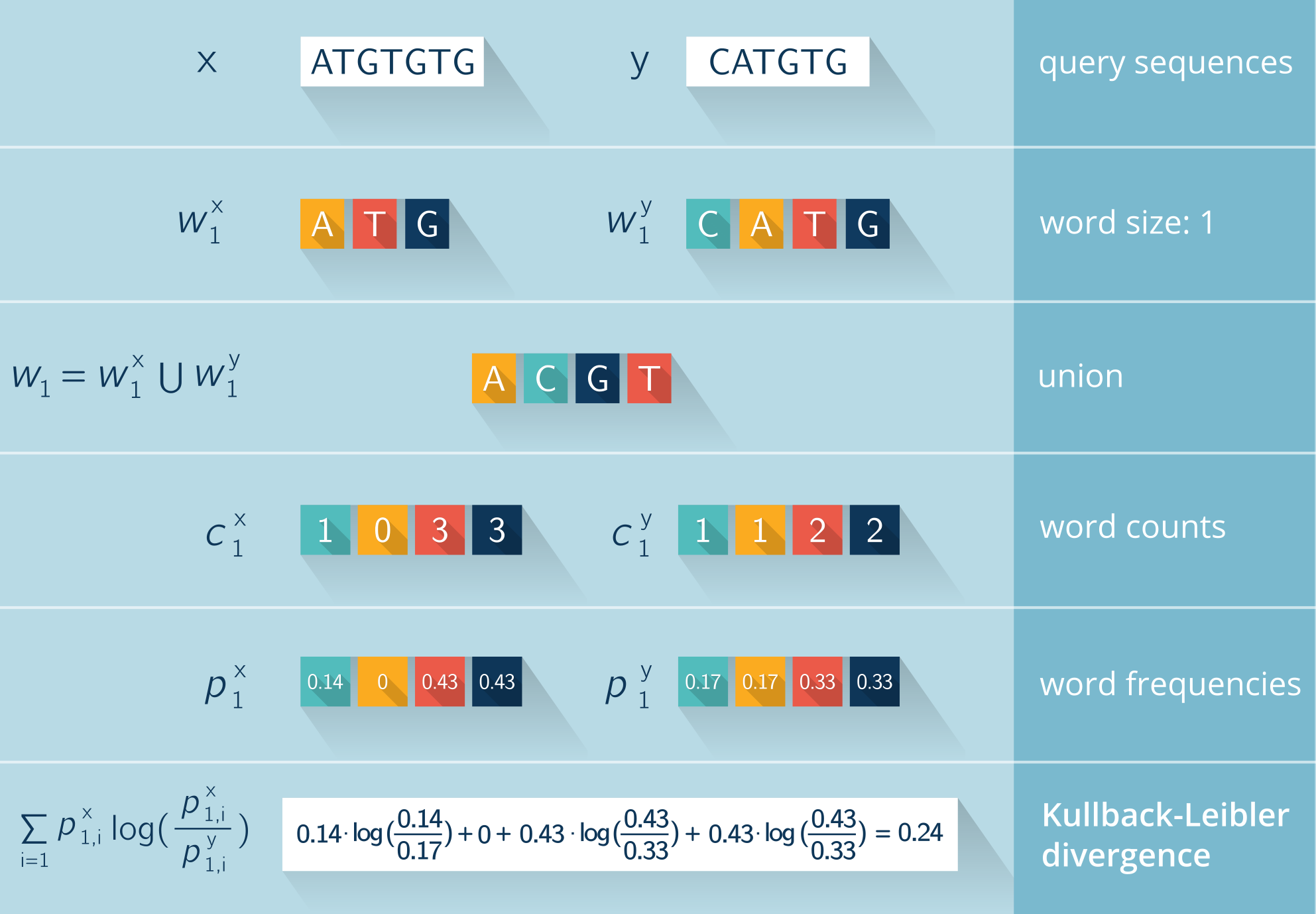


**Fig. S1** Step-by-step calculation of the Kullback–Leibler divergence based on nucleotide frequency vectors


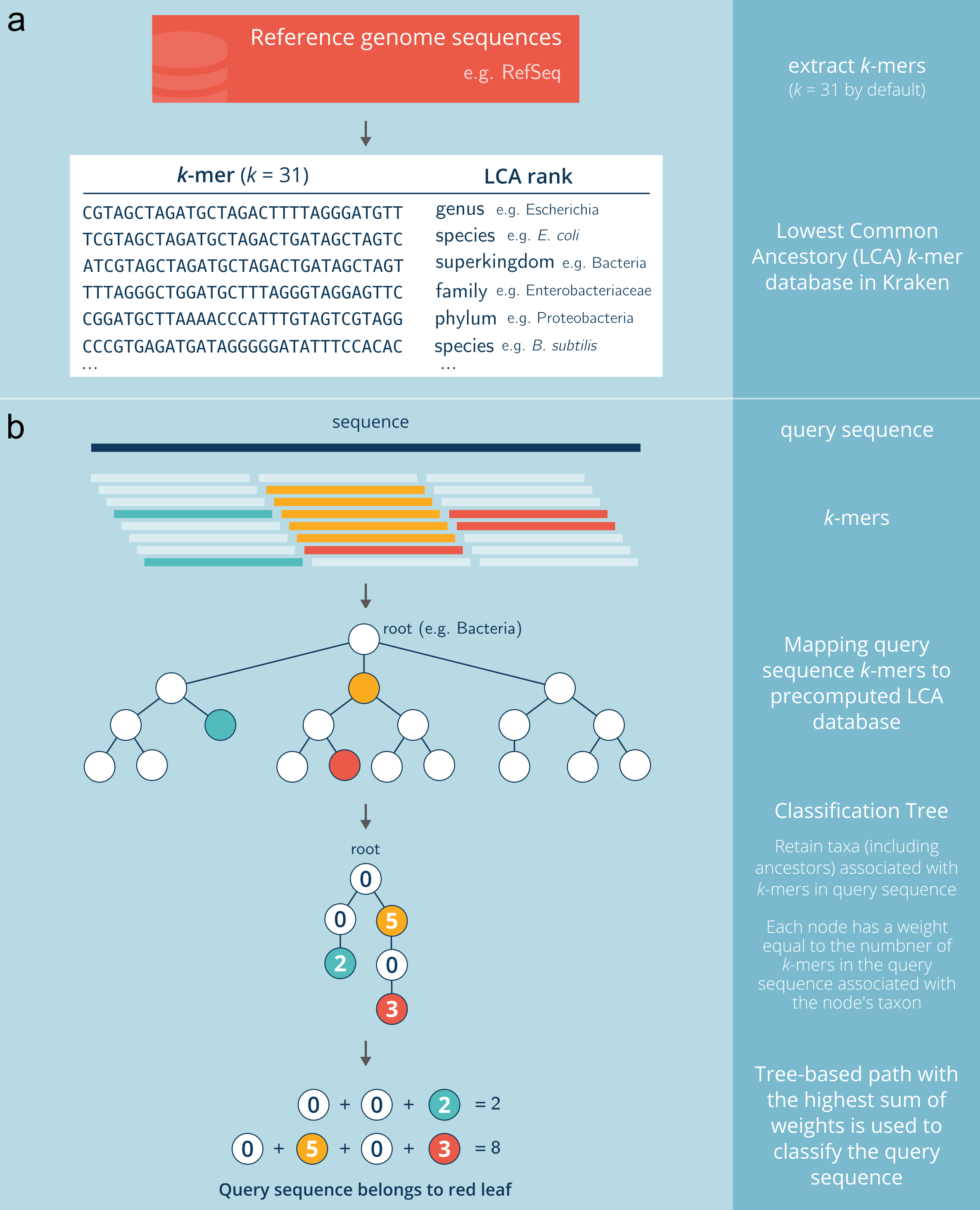


**Fig. S2** Kraken algorithm for taxonomic labeling of metagenomic DNA sequences (based on [83]). **a** Building lowest common ancestor (*LCA*) database from *k*-mers (*k* = 31, by default) extracted from user-specific library of reference genome sequences (e.g. RefSeq). Database contains records consisting of a *k*-mer and the LCA of all descent organisms whose genomes contain that *k*-mer. For example, first *k*-mer record in the table is present in genomes of all species of certain genus (e.g. *Escherichia*) and second *k*-mer is found only in genomes of *E. coli* species. **b** Taxonomic classification of query sequence. Kraken classifies query sequences (e.g. reads) by breaking each into overlapping *k*-mers. Each *k*-mer is mapped to the LCA based on the precomputed *k*-mer database from **a**. A classification tree for a query sequence is found by pruning the taxonomy and only retaining taxa (including ancestors) associated with *k*-mers in that query sequence. In the classification tree, each node has weight equal to the number of *k*-mers in the sequence associated with the node's taxon. The path from root to leaf with the highest sum of weights is used to classify the read. The leaf of this classification path (*red node*) is the classification used to the query sequence
