# Supplementary material for: Alignment-free sequence comparison: benefits, applications, and tools
Source: Genome Biol. 2017 Oct 3;18:186. doi: 10.1186/s13059-017-1319-7 (PMC5627421; doi:10.1186/s13059-017-1319-7)
Supplement: Supplementary file 2 — Ranking list of alignment-free methods and the Smith-Waterman algorithm based on the area under the curve measures across four structural levels of the SCOP2 database. (DOCX 39 kb) [file 13059_2017_1319_MOESM2_ESM.docx]

Additional file 2

**How to compare sequences without performing alignment: a primer**

**Supplementary Table 1** Ranking list of alignment-free methods and the Smith–Waterman algorithm based on the area under the curve measures across four structural levels of the SCOP2 database

| **Distance** | **Reference** | **Word size** | **Vector** | **Additional parameter** | **AUC^a^** | | | | |
| --- | --- | --- | --- | --- | --- | --- | --- | --- | --- |
|  |  |  |  |  | **Class** | **Fold** | **Super family** | **Family** | **Mean↓** |
| d^Normalized Google Dist.^ | [1] | 1 | c | – | 0.63 | 0.78 | 0.80 | 0.84 | 0.760 |
| d^Bray-Curtis^ | [2] | 1 | c | reduced_alph | 0.63 | 0.77 | 0.80 | 0.84 | 0.760 |
| d^Eseq1^ | [3, 4] | 1 | c | reduced_alph | 0.60 | 0.77 | 0.78 | 0.82 | 0.744 |
| d^Eseq2^ | [3, 4] | 1 | c | reduced_alph | 0.61 | 0.75 | 0.77 | 0.81 | 0.733 |
| d^Canberra^ | [2] | 1 | c_w_ | – | 0.62 | 0.73 | 0.76 | 0.79 | 0.725 |
| Smith–Waterman | [5] | – | – | – | 0.62 | 0.67 | 0.78 | 0.81 | 0.720 |
| d^LZ^_*_ | [6] | – | – | – | 0.60 | 0.73 | 0.74 | 0.78 | 0.713 |
| d^S^ | [7] | 1 | f | – | 0.63 | 0.70 | 0.74 | 0.75 | 0.709 |
| d^E^ | [8, 9] | 1 | c | reduced_alph | 0.57 | 0.73 | 0.73 | 0.77 | 0.700 |
| d^Minkowski^ | [10] | 1 | f_std_ | – | 0.63 | 0.64 | 0.70 | 0.70 | 0.668 |
| d^abs_mean^ | [3, 4] | 3 | f | – | 0.62 | 0.64 | 0.70 | 0.71 | 0.668 |
| d^Manhattan^ | [10] | 3 | f | – | 0.62 | 0.64 | 0.70 | 0.71 | 0.668 |
| d^RTD^ | [11] | 1 | c | Google | 0.61 | 0.65 | 0.70 | 0.71 | 0.666 |
| d^abs_mult1^ | [3, 4] | 1 | c | – | 0.55 | 0.70 | 0.68 | 0.72 | 0.663 |
| d^abs_mult2^ | [3, 4] | 1 | c | – | 0.55 | 0.70 | 0.68 | 0.72 | 0.661 |
| d^Chebyshev^ | [2] | 1 | f_stdw_ | – | 0.63 | 0.63 | 0.69 | 0.69 | 0.660 |
| d^LZ^_**1_ | [6] | – | – | – | 0.57 | 0.67 | 0.69 | 0.71 | 0.659 |
| d^LCC^ | [12] | 1 | f_stdw_ | – | 0.59 | 0.63 | 0.68 | 0.70 | 0.648 |
| d^W^ | [13] | – | – | BLOSUM55 | 0.60 | 0.61 | 0.68 | 0.70 | 0.648 |
| d^abs_mult^ | [3, 4] | 1 | f_std_ | – | 0.60 | 0.62 | 0.67 | 0.68 | 0.643 |
| d^LZ^_*1_ | [6] | – | – | – | 0.56 | 0.65 | 0.66 | 0.69 | 0.640 |
| d^EVOL1^ | [14–16] | 1 | f_stdw_ | – | 0.60 | 0.61 | 0.67 | 0.68 | 0.640 |
| d^EVOL2^ | [17] | 1 | f_stdw_ | – | 0.60 | 0.61 | 0.67 | 0.68 | 0.640 |
| d^FFP^ | [18] | 1 | f | reduced_alph | 0.59 | 0.61 | 0.67 | 0.68 | 0.638 |
| d^Sorensen-Dice^ | [2] | 3 | f_boolean_ | – | 0.60 | 0.60 | 0.67 | 0.67 | 0.637 |
| d^Jaccard^ | [2] | 3 | f_boolean_ | – | 0.60 | 0.60 | 0.67 | 0.67 | 0.637 |
| d2 | [19] | 3 | c | – | 0.53 | 0.67 | 0.64 | 0.68 | 0.631 |
| d^KL^ | [20] | 1 | f | – | 0.59 | 0.60 | 0.66 | 0.66 | 0.628 |
| d^Hamming^ | [21] | 2 | f_boolean_ | – | 0.52 | 0.65 | 0.63 | 0.67 | 0.619 |
| d^LZ^ | [6] | – | – | – | 0.49 | 0.62 | 0.58 | 0.61 | 0.575 |
| d^BBC^ | [22] | – | – | *k* = 15 | 0.60 | 0.53 | 0.59 | 0.58 | 0.574 |
| d^NCD^ | [23] | – | – | – | 0.48 | 0.60 | 0.56 | 0.58 | 0.555 |
| d^CV^ | [17] | 3 | c | – | 0.50 | 0.53 | 0.55 | 0.57 | 0.538 |
| d^LZ^_1_ | [6] | – | – | – | 0.44 | 0.55 | 0.49 | 0.52 | 0.499 |

^a^Structural levels in SCOP: *class* proteins with similar secondary structure composition, but different sequences and overall tertiary structures, *folds* protein domains of similar topology and structure without detectable sequence similarity, *superfamilies* similar structures with weak sequence similarity, *families* in cases in which sequence similarity is readily detectable

*AUC* area under the curve, *c* couns, *c_w_* weighted counts, *f* relative frequencies, *f_std_* standardized frequencies with equal aa probabilities, *f_stdw_* standardized frequencies with a priori aa probabilities, *f_w_* weighted frequencies, *reduced_alph* reduced amino acid alphabet to 11 characters

**References**

1. Lee JC, Rashid NA. Adapting normalized google similarity in protein sequence comparison. Proc Int Symp Inf Technol. 2008, ITSim. 2008;1.

2. Jones E, Oliphant T, Pearu P. SciPy: Open source scientific tools for Python. 2001. <http://www.scipy.org/>. Accessed 23 August 2017.

3. Höhl M, Rigoutsos I, Ragan MA. Pattern-based phylogenetic distance estimation and tree reconstruction. Evol Bioinform Online. 2006;2:359–75.

4. Höhl M, Ragan MA. Is multiple-sequence alignment required for accurate inference of phylogeny? Syst Biol 2007;56:206–21.

5. Smith TF, Waterman MS. Identification of common molecular subsequences. J Mol Biol 1981;147:195–7.

6. Otu HH, Sayood K. A new sequence distance measure for phylogenetic tree construction. Bioinformatics. 2003;19:2122–30.

7. Vinga S, Almeida J. Alignment-free sequence comparison—a review. Bioinformatics. 2003;19:513–23.

8. Blaisdell BE. A measure of the similarity of sets of sequences not requiring sequence alignment. Proc Natl Acad Sci U S A. 1986;83:5155–9.

9. Blaisdell BE. Effectiveness of measures requiring and not requiring prior sequence alignment for estimating the dissimilarity of natural sequences. J Mol Evol. 1989;29:526–37.

10. van Helden J. Metrics for comparing regulatory sequences on the basis of pattern counts. Bioinformatics. 2004;20:399–406.

11. Kolekar P, Kale M, Kulkarni-Kale U. Alignment-free distance measure based on return time distribution for sequence analysis: Applications to clustering, molecular phylogeny and subtyping. Mol Phylogenet Evol. 2012;65:510–22.

12. Petrilli P. Classification of protein sequences by their dipeptide composition. Bioinformatics. 1993;9:205–9.

13. Vinga S, Gouveia-Oliveira R, Almeida JS. Comparative evaluation of word composition distances for the recognition of SCOP relationships. Bioinformatics. 2004;20:206–15.

14. Berry MW, Drmac Z, Jessup ER. Matrices, vector spaces, and information retrieval. SIAM Rev. 1999;41:335–62.

15. Stuart GW, Moffett K, Baker S. Integrated gene and species phylogenies from unaligned whole genome protein sequences. Bioinformatics. 2002;18:100–8.

16. Stuart GW, Moffett K, Leader JJ. A comprehensive vertebrate phylogeny using vector representations of protein sequences from whole genomes. Mol Biol Evol. 2002;19:554–62.

17. HAO B, QI J. Prokaryote phylogeny without sequence alignment: from avoidance signature to composition distance. J Bioinform Comput Biol. 2004;2:1–19.

18. Sims GE, Jun S, Wu GA, Kim S. Alignment-free genome comparison with feature frequency profiles (FFP) and optimal resolutions. Proc Natl Acad Sci U S A. 2009;106:2677–82.

19. Song K, Ren J, Reinert G, Deng M, Waterman MS, Sun F. New developments of alignment-free sequence comparison: Measures, statistics and next-generation sequencing. Brief Bioinform. 2014;15:343–53.

20. Wu T-J, Hsieh Y-C, Li L-A. Statistical measures of DNA sequence dissimilarity under Markov chain models of base composition. Biometrics. 2001;57:441–8.

21. Pilcher CD, Wong JK, Pillai SK. Inferring HIV transmission dynamics from phylogenetic sequence relationships. PLoS Med. 2008;5:e69.

22. Liu Z, Meng J, Sun X. A novel feature-based method for whole genome phylogenetic analysis without alignment: Application to HEV genotyping and subtyping. Biochem Biophys Res Commun. 2008;368:223–30.

23. Li M, Chen X, Li X, Ma B, Vitanyi PMB. The similarity metric. IEEE Trans Inf Theory. 2004;50:3250–64.
