# Supplementary material for: Alignment-free sequence comparison: benefits, applications, and tools
Source: Genome Biol. 2017 Oct 3;18:186. doi: 10.1186/s13059-017-1319-7 (PMC5627421; doi:10.1186/s13059-017-1319-7)
Supplement: Supplementary file 3 — Supplementary methods. (DOCX 37 kb) [file 13059_2017_1319_MOESM3_ESM.docx]

Additional file 3

**How to compare sequences without performing alignment: a primer**

**Supplementary methods**

**Benchmark overview**

The performance of 33 alignment-free measures was assessed by calculating areas under the ROC curves (AUC) with reference to the SCOPe database (Structural Classification Of Proteins) [1]. The implementations of the alignment-free methods used in this study are available as a Python package [2]. The accuracy and speed of alignment-free measures were also tested against the Smith–Waterman algorithm implemented in the *water* program (from EMBOSS package [3]).

**Reference SCOPe/ASTRAL dataset**

SCOPe database provides a structural classification of proteins at four levels: class, folds, superfamilies and families. This hierarchical description of proteins allows the evaluation of each method for different levels of sequence similarity. The reference protein dataset used for evaluating the accuracy of each sequence comparison method was constructed based on the SCOPe database 2.06, called ASTRAL 2.06, which includes all SCOPe sequences that share less than 40% identity to each other. This data set is considered to be one of the most reliable benchmark test sets in the evaluation of different methods to detect remote protein homologies.

Similar to the study by Vinga et al. 2004 [4], the original ASTRAL 2.06 dataset was trimmed to create a reference data set for the benchmark (Table 1, below). This data set:

- excludes sequences with ambiguous amino acids
- excludes families with less than five proteins
- includes only the four major classes
- α class: comprising mainly of proteins with a α helix
- β class: proteins formed essentially by β-sheet structures
- α/β class :proteins with mixtures of α-helices and β-strands
- α + β class: those where α-helices and β-strands are largely segregated.

**Table 1** Reference sequence set used in the benchmark

| Dataset | α class | | | | β class | | | | α/β class | | | | α + β class | | | | Total |
| --- | --- | --- | --- | --- | --- | --- | --- | --- | --- | --- | --- | --- | --- | --- | --- | --- | --- |
|  | pr | fa | sf | cf | pr | fa | sf | cf | pr | fa | sf | cf | pr | fa | sf | cf |  |
| astral40 | 2439 | 984 | 513 | 289 | 2795 | 890 | 365 | 177 | 3970 | 939 | 246 | 148 | 3346 | 1217 | 561 | 385 | 12,550 |
| Reference | 1072 | 98 | 57 | 46 | 1469 | 112 | 62 | 44 | 2478 | 159 | 75 | 59 | 1577 | 144 | 88 | 70 | 6596 |

Number of sequences: proteins (*pr*), families (*fa*), superfamilies (*sf*) and folds (*cf*)

The reference data set is available for download [5].

**Benchmark procedure**

The 33 alignment-free methods were used to calculate the dissimilarity/distance measures between all 21,750,310 possible combination pairs of 6596 proteins from the reference dataset. Different combinations of input parameters were used to run word-based methods (i.e. word size from 1 to 4; protein alphabet consisted of either the original 20 amino acids or 11 reduced alphabet: different vector types such as count occurrences, frequencies, weighted counts, weighted frequencies, standardized frequencies with equal/equilibrium amino acid frequencies) and W-metric (different substitution matrices: PAM120-PAM250, BLOSUM30-70). In total, 529 independent runs of alignment-free calculations were performed using the reference dataset as query. The Smith–Waterman algorithm (*water*) was run using BLOSUM50/BLOSUM40 scoring matrices and default parameters for gap scoring (i.e. gap open: 10, gap extend: 0.5).

For each alignment-free method run, the distances between all protein pairs were subsequently sorted, from maximum to minimum similarity. The comparative test procedure was based on a binary classification of each protein pair, where 1 corresponds to the two proteins sharing the same group in the SCOP database, 0 otherwise. Since the group can be defined at any one of the four different levels of the database (family, superfamily, fold, class), each protein pair was associated to four binary classifications (one for each level). The similarity measure for alignment method was based on the Smith–Waterman score, with no correction for statistical significance.

R package ROCR [6] was used to obtain ROC curves and AUC values for the Smith–Waterman run and all alignment-free methods (at each SCOP level).

**References**

1. Fox NK, Brenner SE, Chandonia J-M. SCOPe: Structural Classification of Proteins--extended, integrating SCOP and ASTRAL data and classification of new structures. Nucleic Acids Res. 2014;42:D304-9.

2. Python package. <https://github.com/aziele/alfpy>. Accessed 23 August 2017.

3. Rice P, Longden I, Bleasby A. EMBOSS: the European Molecular Biology Open Software Suite. Trends Genet. 2000;16:276–7.

4. Vinga S, Gouveia-Oliveira R, Almeida JS. Comparative evaluation of word composition distances for the recognition of SCOP relationships. Bioinformatics. 2004;20:206–15.

5. <http://www.combio.pl/alfree/download/data/>. Accessed 23 August 2017.

6. Sing T, Sander O, Beerenwinkel N, Lengauer T. ROCR: visualizing classifier performance in R. Bioinformatics. 2005;21:3940–1.
